# Differential Expression of mRNA Encoding Cytokines and Chemokines in the Reproductive Tract after Infection of Mice with *Chlamydia trachomatis*

**DOI:** 10.4172/2161-038X.1000152

**Published:** 2015-09-09

**Authors:** Katheryn L. Cerny, Maranda Van Fleet, Anatoly Slepenkin, Ellena M. Peterson, Phillip J. Bridges

**Affiliations:** 1 Department of Animal and Food Sciences, University of Kentucky, Lexington, KY, USA; 2 Department of Pathology and Laboratory Medicine, University of California Irvine, Irvine, CA, USA

**Keywords:** *Chlamydia trachomatis*, Oviduct, Inflammation, Cytokines, Chemokines, Immune response

## Abstract

Infection with *Chlamydia trachomatis* targets epithelial cells within the genital tract which respond by secreting chemokines and cytokines. Persistent inflammation can lead to fibrosis, tubal infertility and/or ectopic pregnancy; many infections are asymptomatic. Most studies have investigated the inflammatory response in the initial stages of infection, less is known about the later stages of infection, especially with a low, potentially asymptomatic, bacterial load. Our objective was to determine the inflammatory mediators involved in clearance of low-grade infection and the potential involvement in chronic inflammation. Six to eight week old C3H/HeJ mice were pretreated with 2.5 mg medroxyprogesterone acetate on day -10 and -3 before infection. Mice (n=3 for 28 d, n=3 for 35 d) were infected with 5 × 10^2^ inclusion-forming units of *C. trachomatis*, serovar D; vaginal cultures were obtained weekly to monitor infection. Control mice (n=3 for 28 d, n=3 for 35 d) were sham infected. Mice were killed on day 28 (experiment 1) and day 35 (experiment 2) post-infection and vaginal tissue, uterine horns and oviducts collected for analysis of mRNAs encoding inflammatory cytokines and chemokines. Total RNA was isolated and a superarray analysis performed using mouse Cytokines and Chemokines PCR arrays (Qiagen, Valencia, CA). Statistical differences in gene expression were determined using a paired Students t-test. At 28 days after infection, the expression of mRNA encoding 6, 35 and 3 inflammatory genes differed from controls in vaginal, uterine and oviductal tissues, respectively (P<0.05). At 35 days after infection, the expression of mRNA encoding 16, 38 and 14 inflammatory genes differed from controls in vaginal, uterine and oviductal tissues, respectively (P<0.05). Understanding the mechanisms involved in the inflammatory response at later stages of infection should aid in the development of treatment options that minimize the development of asymptomatic, chronic inflammation-induced infertility.

## Introduction

*Chlamydia trachomatis* is an obligate intracellular pathogen and the most frequently reported sexually transmitted bacteria in the United States [1]. *C. trachomatis* targets epithelial cells within the genital tract initiating an immune response. Infectious load is correlated to clinical pathogenesis [2,3]; infection with *C. trachomatis* is often asymptomatic. If left untreated the bacteria can ascend to and infect the oviducts [4,5]. Untreated *C. trachomatis* infection can lead to persistent or recurrent inflammation, fibrosis, scarring, pelvic inflammatory disease (PID), tubal infertility, and/or an increased susceptibility to ectopic pregnancy [6,7].

Upon infection, *C. trachomatis* elementary bodies (EBs) invade host epithelial cells in the genital tract. Within the host cells, EBs differentiate into reticulate bodies (RBs) which actively replicate within the host cell cytoplasm and then reorganize back into infectious EBs. This biphasic life cycle as well as adaptation to evade the immune response allows *C. trachomatis* to persist for extended periods within host epithelial cells, inducing a chronic inflammatory response [8–12].

Previous studies have investigated the inflammatory response of *C. trachomatis* in the initial stages of infection, including regulation by cytokines, chemokines and inflammatory mediators involved in the recruitment of immune cells [5,8,10,13,14]. For example, Rasmussen et al. [10] demonstrated that once *C. trachomatis* has established infection within epithelial cells, the innate immune response allows for the production of pro-inflammatory cytokines such as interleukins 1, 6, 8 (Il-1, Il-6, Il-8), tumor necrosis factor-alpha (TNF-α), and colony stimulating factor 2 (CSF 2). Secretion of these cytokines and chemokines recruit immune cells such as natural killer (NK) cells and phagocytes. Following an established intracellular infection, the T-cell mediated immune response then becomes the critical element required for clearance [15]. However, evidence suggests that this T-cell response also contributes to the pathology following infection. Th1 cells limit replication of *C. trachomatis*, but Th2 cells inhibit Th1 responses leading to continued production of pro-inflammatory cytokines which can lead to fibrosis [16]. *C. trachomatis* also induces production of TNF-α, which promotes apoptosis of infected and bystander cells [17]. Overall, understanding cytokine and chemokine regulation during both acute and chronic phases of infection may contribute to the development of treatment options that will minimize the long-term inflammatory consequences attributed to this disease.

Limited investigation of the overall inflammatory response during the later stages of infection has been performed, especially after infection with a low bacterial load. For example, Maxion and Kelly [5] used 10^7^ IFUs of the mouse pneumonitis biovar of *C. trachomatis* and reported that cytokine and chemokine expression differs in anatomically distinct regions of the genital tract. Specifically, these authors investigated the expression of chemokines associated with Th1 and Th2 responses in the oviducts and cervical-vaginal regions of the reproductive tract during the induction phase (0–14 days) and resolution phase (14–35 days) of infection. Our objective was to determine within the reproductive tract the concurrent level of expression of mRNA encoding inflammatory mediators during the later phases of infection using a relatively low infectious load of *C. trachomatis* biovar, serovar D, one of the most prevalent serovars involved in urogenital infections of humans [18]. Two separate experiments were performed, with tissues collected at 4 and 5 weeks after infection (experiment 1 and 2, respectively). Our hypothesis was that mRNA encoding pro-inflammatory cytokines and chemokines will be differentially expressed in the female reproductive tract of mice infected with *C. trachomatis* at both 28 and 35 days post-infection compared to controls.

## Materials and Methods

### Ethics statement

All animal experiments were performed according to the guidelines and protocol approved by the University of California Irvine Institutional Animal Care and Use Committee (protocol # 2009–2868).

### Animal model

Using a previously described model of confirmed genital infection by *C. trachomatis*, serovar D, female C3H/HeJ mice, 6- to 8-week old, (Jackson Laboratories, Sacramento, CA) were pretreated with 2.5 mg medroxyprogesterone acetate (SICOR Pharmaceuticals) on Days -10 and -3 before infection [19,20]. In both experiments mice were infected via vaginal challenge with 5 × 10^2^ inclusion-forming units (IFUs) of *C. trachomatis*, serovar D in 0.01 mL of Eagle Minimal essential media (MEM, Gibco) on Day 0, as previously described [19,20]. Control mice were also pretreated with medroxyprogesterone acetate, but were sham infected with Eagle Minimal essential media (MEM, Gibco) alone. Vaginal swabs were obtained twice weekly after infection and cell cultures were performed to monitor infection as previously described [19–21].

Mice were killed on day 28 (n=3 for control and infected) in experiment 1 and day 35 (n=3 for control and infected) in experiment 2. Immediately before being sacrificed vaginal cultures were obtained and all mice inoculated vaginally with *C. trachomatis* remained culture positive but at a significantly lower level than that obtained throughout the first two weeks of infection. Results of vaginal cultures following infection with this strain/dose of *C. trachomatis*, serovar D have been reported, including number of IFUs recovered [20]. Vaginal tissue, uterine horns and the oviducts were collected and snap-frozen for later extraction of RNA.

### Isolation of RNA and gene expression analysis

Total RNA was extracted from each tissue sample using TRIzol reagent (Invitrogen, Carlsbad, CA) and purified through RNeasy columns (Qiagen, Valencia, CA). To determine the effect of treatment on the expression of genes involved in the inflammatory response, a targeted real time PCR SuperArray analysis was performed using RT2 Profiler PCR arrays for mouse Cytokines and Chemokines (Qiagen), as previously described [22]. Real-time PCR were performed on an Eppendorf Mastercycler ep realplex2 system (Eppendorf, Hamburg, Germany).

Gene expression was standardized against GAPDH as a housekeeping gene and analyzed by the 2−ΔΔCT method [23]. Statistical differences in the expression of mRNA were determined using a paired Students t-test.

## Results

### Experiment 1: Expression of mRNA encoding inflammatory genes in vaginal, uterine and oviduct tissues at 28 days post-infection

Gene expression analysis was used to determine the effect of infection on the expression of inflammatory mRNAs at 28 days post-infection. In vaginal tissue collected at 28 days after infection, the expression of mRNA encoding 6 inflammatory genes increased and no genes decreased when compared to controls (Table 1).

In uterine samples collected at 28 days after infection, the expression of mRNA encoding 32 inflammatory genes increased and 3 genes decreased when compared to controls (Table 2).

Of the 6 inflammatory mRNAs that increased within vaginal tissue after infection, 4 were also differentially affected by treatment in uterine samples. In oviducts collected at 28 days post-infection, the expression of mRNA encoding 2 inflammatory genes increased and 1 gene decreased. Of the 3 inflammatory genes affected by treatment within the oviduct, mRNA encoding 1 gene, chemokine (c-c motif) ligand 12 (Ccl12), was also increased in uterine samples.

### Experiment 2: Expression of mRNA encoding inflammatory genes in vaginal, uterine and oviduct tissues at 35 days post-infection

In vaginal tissue collected 35 days after infection, the expression of mRNA encoding 8 inflammatory genes was increased and 8 decreased when compared to controls (Table 1). In uterine samples collected at 35 days after infection, the expression of mRNA encoding 32 inflammatory genes increased and 6 genes decreased compared to controls (Table 3).

Of the 16 inflammatory mRNAs affected by treatment in vaginal tissue, 7 were also differentially expressed in uterine samples and 3 in oviduct samples. In oviducts collected at 35 days post infection, the expression of mRNA encoding 13 inflammatory genes was increased and 1 gene decreased (Table 4).

Of the 14 inflammatory mRNAs affected by treatment within the oviduct, 5 were also differentially expressed in uterine samples and 3 in vaginal tissue.

## Discussion

The host response to infection with *C. trachomatis* includes the induction of pro inflammatory cytokines and chemokines which leads to innate and adaptive immune cell recruitment and activation [24]. Although the immune response is critical to the clearance of infection, the cellular immune response in particular can cause tissue damage that promotes fibrosis and can lead to infertility [16]. Considering that infection with *C. trachomatis* is often asymptomatic, the objective of these two experiments was to identify inflammatory mediators induced during the later phases of low-dose *C. trachomatis* genital infection in order to advance our understanding of disease progression and the immune response involved in potentially asymptomatic chronic inflammation.

It is well known that susceptibility to *C. trachomatis* infection is genetically controlled in mice. Both Tuffrey et al. [25–27] and Peterson et al. [19–21] have shown that human serovars of *C. trachomatis* can infect the genital tract of mice, specifically C3H/HeJ mice. Progesterone pretreatment is necessary, but this strain of mice remains culture positive for more than 4 weeks following infection [20]. In the current study, infected mice continued to have positive vaginal *C. trachomatis* cultures for the duration of the experiments with IFUs lower when the mice were killed compared to the first two weeks of infection.

In experiment 1, mice were killed 28 days post-infection. In vaginal tissue, mRNA encoding several cytokines and chemokines was affected by infection with C. trachomatis. Among differentially-regulated genes in vaginal samples, the expression of mRNA encoding chemokine (c-c motif) ligand 4 (Ccl4), also known as macrophage inflammatory protein-1β (MIP-1β), a potent lymphocyte chemo attractant, was induced at 28 days of infection, with greater than a 6-fold increase compared to controls. C-C motif chemokines are a subgroup of chemokines with two adjacent cysteine residues near the amino terminus [28]. Yilma et al. [29] reported an increase in Ccl4 production in mouse macrophages during the early response to C. trachomatis infection; therefore, our results suggest that this cytokine is actively involved in both the early response and late phases of infection. Interestingly, Ccl4 is highly related to macrophage inflammatory protein-1α (Ccl3) and it is thought that these C-C motif chemokines are secreted to recruit specific T cell subsets during the immune response [30,31]. In our study, expression of mRNA encoding Ccl3 in the vagina was also increased at 28 days post-infection.

Of all the differentially affected mRNAs in vaginal tissue, the largest fold-change in 28 day infected samples was seen in the induction of mRNA encoding CD40 ligand (Cd40lg). Cd40lg is mostly found on the surface of CD4+ T cells and its interaction with Cd40 is required in the activation of humoral and cellular immune responses [32].

Within the uterus, treatment affected the expression of mRNA for five C-X-C motif chemokines. C-X-C motif chemokines are a subgroup of chemokines that have amino terminus cysteine residues separated by one amino acid [28]. Most notable is the induction of mRNA encoding chemokine (c-x-c motif) ligand 9 (Cxcl9). Previous studies have reported that Cxcl9 peaks during the early phases of infection in the upper genital tract and may be involved in Th1 responses [5], our results suggest that within the uterus, Cxcl9-mediated inflammation remains ongoing even after the initial phase of infection. The expression of mRNA encoding several interleukins was also induced in the infected mouse uterus at 28 days post-infection, including Il1b, Il12a, Il12b, Il16, Il18, and Il27. Notably, Il12 is also reported to be involved in Th1 responses [33]. In our results, mRNA for Il12 subunit alpha (Il12a) and subunit beta (Il12b) was induced at 28 days after infection. Several tumor necrosis factor family members and interferons were also induced, supporting the hypothesis that inflammation remains active and ongoing within the uterus during the late, resolution phase of infection.

When compared to the response observed in vaginal and uterine tissues at 28 days, the oviduct had fewer mRNA differentially expressed after infection of mice with *C. trachomatis*.

The expression of mRNA encoding two genes was increased and one gene decreased. *C. trachomatis* -induced cell death within the oviduct is of concern due to long term sequelae, especially when considering that upon initial intracellular invasion of epithelial cells, *C. trachomatis* has the ability to prevent apoptosis of infected cells, therefore promoting infection [8,13,14]. Interestingly, the expression of mRNA encoding fas ligand (TNF superfamily, member 6; Fasl), a key mediator of apoptosis, was not affected by treatment at 28 days post infection within the oviduct.

In experiment 2, mice were killed 35 days post-infection. The level of mRNA encoding 4 interleukins (Il10, Il11, Il1rn, Il1a) was increased and 1 interleukin (Il18) decreased in vaginal tissue collected at 35 days post-infection. Of these, Il18 is reported to interact with Il12 to stimulate interferon gamma (IFN-γ) production from NK cells during the early host response to infection [34]. Although studies have reported that IFN-γ is crucial for immune cell responses to *C. trachomatis* [34–36], the expression of mRNA for Il12 did not differ at 35 days after infection and there was a decrease in levels of mRNA for Il18. Furthermore, IFN-γ had the greatest fold change of all differentially induced mRNA in the infected vaginal tissues, suggesting that IFN-γ production is being stimulated by other immunoregulatory factors at this later stage of infection.

The increase in expression of mRNA encoding Il10 within vaginal samples collected at 35 days after infection was not expected. Interleukin-10 is considered an anti-inflammatory cytokine and a recent study using *C. trachomatis* infected HeLa cells demonstrated that exogenous Il10 treatment decreased several inflammatory cytokines including TNF [37,38].

Similar to vaginal tissues, uterine samples collected 35 days post-infection with *C. trachomatis* had increased expression of Il10 and TNF. Furthermore, an increase in adiponectin (Adipoq) was observed. Similar to Il10, Adipoq has anti-inflammatory properties including regulating cell defense and survival during stress conditions [39,40]. In addition, a dramatic increase in the expression of mRNA for Cxcl11 (102-fold change) was observed. Cxcl11 shares features with Cxcl9 and Cxcl10, including induction by interferons and expression on activated Th1 cells [41,42]. It is reported that the Th1 response is crucial for controlling *C. trachomatis* infection, our results that mRNA for these transcripts were induced within the infected uterus is therefore consistent with other studies.

Within the oviduct mRNA encoding 13 genes was increased and one gene decreased at 35 days after infection. The expression of mRNA encoding fasl increased at 35 days after infection with a 15-fold-change. In addition, changes in mRNA for other inflammatory mediators involved in tissue damage were detected, including Il1-a, which is released from lysed cells and acts by stimulating further cytokine release from neighboring cells [10]. Interestingly, levels of mRNA for Leukemia inhibitory factor (Lif) were increased in 35 day infected oviducts. Guney et al. [43] demonstrated that LIF expression is increased in the oviducts of woman with ectopic pregnancies compared to non-pregnant woman. Furthermore, Ji et al. [44] proposed that LIF facilitates implantation of the embryo in the oviduct when the stromal surface is exposed due to epithelial cell shedding caused by chronic inflammation. The results shown here warrant further investigation especially since *C. trachomatis* have the ability to not only disrupt infected epithelial cells, but also non-infected cells in proximity to the infection [8]. In oviductal epithelia, these changes in gene expression and disruption of cellular processes can increase the risk of chronic inflammation-induced pelvic inflammatory disease and infertility.

Overall, this study examined the coordinated and concurrent expression of mRNA encoding multiple cytokines in spatially distinct sections of the reproductive tract. We investigated the later stages of infection using a relatively low infectious dose in order to obtain a better understanding of the genetic mechanisms involved in chronic inflammation and cellular damage. Differences in the magnitude of response to infection in differing regions of the reproductive tract were expected, as were differences in the level of expression of specific mRNAs within a tissue over time [5], illustrating well the dynamic nature of the inflammatory response to infection and the need for inclusive analyses of inflammatory mediators.

Understanding the mechanisms involved in the inflammatory response at late stages of infection should aid in the development of treatment options that minimize chronic inflammation-induced pelvic inflammatory disease and infertility.

## Figures and Tables

**Table 1:** Effect of treatment on the expression of mRNAs in the vagina at 28 and 35 days post-infection. The normalized average ΔC+ value was calculated using GAPDH as the house-keeping gene. Fold change values (infected over control) in gene expression are presented as average fold change (2^−(average Ct)^) for differentially expressed mRNAs (P<0.05).

28 Day infected vs. control				
Gene Symbol	Control Avg. ΔC+ SEM	Infected Avg. ΔC+ SEM	Fold change	p-value
Ccl24	12.79 ± 0.76	10.62 ± 0.53	4.5	0.007
Ccl3	10.18 ± 0.54	7.81 ± 1.00	5.5	0.025
Ccl4	9.03 ± 0.59	6.28 ± 0.44	6.7	0.036
Cd40lg	14.01 ± 0.30	11.05 ± 0.36	7.8	0.011
Cxcl1	9.00 ± 0.25	6.18 ± 0.24	7.1	0.028
Il22	15.96 ± 0.37	15.03 ± 0.39	1.9	0.024
35 day infected vs. control				
Bmp7	6.43 ± 0.17	6.51 ± 0.25	0.5	0.032
Csf3	4.88 ± 0.05	5.47 ± 0.06	0.5	<0.001
Ccl4	9.03 ± 0.58	6.28 ± 0.23	4.2	0.019
Ctf1	6.67 ± 0.15	7.19 ± 0.12	0.6	0.023
Hprt	1.65 ± 0.14	1.81 ± 0.04	0.6	0.022
Ifna2	11.3 ± 0.19	11.41 ± 0.24	0.4	0.014
Ifng	13.04 ± 0.54	9.68 ± 0.43	8.5	0.035
Il10	9.94 ± 0.38	8.35 ± 0.32	2.8	0.048
Il11	11.76 ± 0.07	10.52 ± 0.23	2.3	0.03
Il18	5.86 ± 0.22	6.54 ± 0.06	0.4	0.014
Il1a	7.13 ± 0.09	5.97 ± 0.04	1.4	0.007
Il1rn	6.36 ± 0.37	5.95 ± 0.13	1.9	0.042
Mif	0.43 ± 0.12	0.76 ± 0.08	0.7	0.045
Pf4	5.15 ± 0.19	5.26 ± 0.06	0.6	0.031
Thpo	12.95 ± 0.6	13.76 ± 0.16	3.7	0.019
Tnf	8.4 ± 0.19	6.86 ± 0.09	2.4	0.002

**Table 2:** Effect of treatment on the expression of mRNAs in the uterus at 28 days post-infection. The normalized average ΔC+ value was calculated using GAPDH as the house-keeping gene. Fold change values (infected over control) in gene expression are presented as average fold change (2^−(average Ct)^) for differentially expressed mRNAs (P<0.05).

28 Day infected vs. control									
Gene Symbol	Control Avg. ΔC + SEM	Infected Avg. ΔC+ SEM	Fold Change	p-value	Gene Symbol	Control Avg. ΔC+ SEM	Infected Avg. ΔC+ SEM	Fold Change	p-value
Ccl1	13.84 ± 0.32	12.03 ± 0.32	3.5	0.04	Il16	7.41 ± 0.17	6.24 ± 0.27	2.2	0.045
Ccl17	9.56 ± 0.11	7.76 ± 0.21	3.5	0.009	Il27	11.83 ± 0.11	9.70 ± 0.15	4.4	0.002
Ccl2	7.31 ± 0.29	4.53 ± 0.35	6.8	0.025	Lta	11.01 ± 0.21	9.01 ± 0.37	4	0.037
Ccl22	9.18 ± 0.13	7.45 ± 0.31	3.3	0.025	Ltb	6.85 ± 0.16	3.58 ± 0.29	9.6	0.009
Ccl24	11.64 ± 0.13	12.81 ± 0.30	0.4	0.018	Tgfb2	0.67 ± 0.04	2.57 ± 0.76	0.3	0.03
Ccl3	11.52 ± 0.34	8.49 ± 0.13	8.2	0.001	Tnf	9.77 ± 0.22	6.93 ± 0.30	7.2	0.013
Ccl4	9.73 ± 0.39	6.41 ± 0.15	10	0.001	Tnfsf11	10.44 ± 0.47	8.68 ± 0.28	3.4	0.029
Ccl5	5.80 ± 0.05	1.75 ± 0.27	16.6	0.008	Xcl1	7.00 ± 0.19	5.29 ± 0.23	3.3	0.016
Cd40lg	14.40 ± 0.61	8.19 ± 0.35	74	0.016	Ccl12	7.01 ± 0.25	3.70 ± 0.30	9.9	0.015
Csf2	12.12 ± 0.45	9.31 ± 0.17	7	0.002	Ccl7	6.68 ± 0.33	3.63 ± 0.36	8.3	0.027
Cxcl10	8.91 ± 0.06	4.99 ± 0.31	15.1	0.012	Cxcl5	10.50 ± 0.10	5.42 ± 0.46	34	0.049
Cxcl13	7.80 ± 0.73	4.10 ± 0.13	13	0.001	Gusb	2.88 ± 0.09	2.27 ± 0.15	1.5	0.035
Cxcl16	5.19 ± 0.15	3.27 ± 0.20	3.8	0.008	Il12a	12.98 ± 0.35	11.79 ± 0.26	2.3	0.045
Cxcl9	8.26 ± 0.21	2.47 ± 0.18	55.3	0.001	Il18	6.87 ± 0.06	5.92 ± 0.11	1.9	0.003
Fasl	10.13 ± 0.21	6.46 ± 0.20	12.8	0.002	Il1b	8.29 ± 0.52	5.55 ± 0.33	6.7	0.023
Ifna2	11.25 ± 0.08	12.23 ± 0.38	0.5	0.041	Osm	10.42 ± 0.13	8.05 ± 0.13	5.2	0.001
Ifng	11.30 ± 0.08	7.21 ± 0.28	17.1	0.006	Tnfsf10	5.96 ± 0.13	4.12 ± 0.29	3.6	0.02
Il12b	10.68 ± 0.24	8.07 ± 0.26	6.1	0.007					

**Table 3:** Effect of treatment on the expression of mRNAs in the uterus at 35 days post-infection. The normalized average ΔC+ value was calculated using GAPDH as the house-keeping gene. Fold change values (infected over control) in gene expression are presented as average fold change (2^−(average Ct)^) for differentially expressed mRNAs (P<0.05).

35 day infected vs. control									
Gene Symbol	Control Avg. ΔC+ SEM	Infected Avg. ΔC+ SEM	Fold change	p-Value	Gene symbol	control Avg. ΔC+ SEM	Infected Avg. ΔC+ SEM	Fold change	p-value
Ccl1	13.26 ± 0.25	11.66 ± 0.25	3	0.016	Il27	12.87 ± 0.30	9.73 ± 0.27	8.8	0.012
Ccl17	9.41 ± 0.15	7.97 ± 0.34	2.7	0.041	Lta	10.67 ± 0.19	9.22 ± 0.05	2.7	<0.001
Ccl2	6.72 ± 0.14	5.44 ± 0.17	2.4	0.007	Ltb	6.92 ± 0.19	4.28 ± 0.09	6.2	<0.001
Ccl22	8.69 ± 0.15	7.22 ± 0.15	2.8	0.005	Tgfb2	1.26 ± 0.14	3.70 ± 1.24	0.2	0.021
Ccl24	11.25 ± 0.08	13.93 ± 0.94	0.2	0.012	Tnf	9.73 ± 0.09	6.98 ± 0.28	6.7	0.013
Ccl3	10.83 ± 0.31	8.35 ± 0.08	5.6	<0.001	Tnfsf11	9.94 ± 0.12	9.06 ± 0.14	1.8	0.013
Ccl4	9.08 ± 0.23	6.26 ± 0.04	7	<0.001	Xcl1	7.12 ± 0.19	5.41 ± 0.40	3.3	0.05
Ccl5	5.47 ± 0.21	2.32 ± 0.09	8.9	<0.001	Adipoq	11.58 ± 1.29	6.81 ± 0.40	27.2	0.034
Cd40lg	13.94 ± 0.93	8.64 ± 0.16	39.4	0.001	Cd70	11.19 ± 0.19	10.38 ± 0.07	1.7	0.008
Csf2	11.51 ± 0.29	8.78 ± 0.16	6.6	0.002	Cntf	7.99 ± 0.08	8.62 ± 0.17	0.6	0.023
Cxcl10	9.18 ± 0.19	5.08 ± 0.27	17.1	0.005	Csf3	7.55 ± 0.15	6.56 ± 0.18	2	0.017
Cxcl13	8.83 ± 0.27	4.51 ± 0.36	20	0.017	Cxcl1	11.41 ± 0.43	7.49 ± 0.46	15.1	0.029
Cxcl16	5.00 ± 0.23	3.14 ± 0.13	3.6	0.002	Cxcl11	12.94 ± 0.27	6.26 ± 0.34	102.5	0.018
Cxcl9	8.06 ± 0.22	2.41 ± 0.26	50.3	0.006	Il10	11.18 ± 0.41	8.77 ± 0.15	5.3	0.003
Fasl	10.25 ± 0.04	6.92 ± 0.29	10.1	0.013	Il21	15.24 ± 0.15	10.55 ± 0.46	25.8	0.047
Ifna2	10.87 ± 0.31	12.36 ± 0.33	0.4	0.037	Il3	15.11 ± 0.11	14.13 ± 0.22	2	0.026
Ifng	10.76 ± 0.16	7.33 ± 0.09	10.8	<0.001	Thpo	11.89 ± 0.16	13.27 ± 0.36	0.4	0.02
Il12b	10.31 ± 0.23	8.46 ± 0.12	3.6	0.002	Tnfsf10	5.82 ± 0.18	3.80 ± 0.19	4	0.004
Il16	7.75 ± 0.11	6.75 ± 0.20	2	0.02	Vegfa	4.23 ± 0.13	4.83 ± 0.09	0.7	0.023

**Table 4:** Effect of treatment on the expression of mRNAs in the oviduct at 28 and 35 days post-infection. The normalized average ΔC+ value was calculated using GAPDH as the house-keeping gene. Fold change values (infected over control) in gene expression are presented as average fold change (2^−(average Ct)^) for differentially expressed mRNAs (P<0.05).

28 Day infected vs. control				
Gene Symbol	Control Avg. ΔC+ SEM	Infected Avg. ΔC+ SEM	Fold change	p-value
Ccl12	6.81 ± 0.37	4.39 ± 0.51	5.3	0.045
Il13	10.45 ± 0.21	11.69 ± 0.28	0.4	0.029
Il23a	11.43 ± 0.10	10.96 ± 0.08	1.4	0.021
**35 Day infected vs. control**				
Bmp7	7.97 ± 0.21	6.99 ± 0.12	2	0.011
Ccl17	7.11 ± 0.52	6.19 ± 0.32	6.1	0.021
Cd40lg	13.02 ± 0.30	10.40 ± 0.36	6.2	0.03
Cx3cl1	5.16 ± 0.21	4.16 ± 0.18	2	0.025
Cxcl1	12.57 ± 0.25	9.99 ± 0.24	6	0.007
Fasl	12.75 ± 0.66	8.79 ± 0.34	15.6	0.019
Gpi1	2.71 ± 0.04	1.56 ± 0.26	2.2	0.039
Hsp90ab1	0.53 ± 0.18	1.98 ± 0.26	2.7	0.027
Il12b	13.10 ± 0.24	10.47 ± 0.17	6.2	0.003
Il1a	11.42 ± 0.22	9.65 ± 0.23	3.4	0.011
Il9	7.16 ± 0.21	8.21 ± 0.26	0.5	0.044
Lif	10.03 ± 0.38	8.77 ± 0.08	2.4	0.016
Mif	1.55 ± 0.09	0.17 ± 0.29	2.6	0.044
Osm	11.50 ± 0.44	9.93 ± 0.33	3	0.047
